# Endoscopy applications for the second law analysis in hydromagnetic peristaltic nanomaterial rheology

**DOI:** 10.1038/s41598-022-04945-1

**Published:** 2022-01-28

**Authors:** Muhammad Awais, Muhammad Shoaib, Muhammad Asif Zahoor Raja, Saba Arif, Muhammad Yousaf Malik, Kottakkaran Sooppy Nisar, Khadiga Ahmed Ismail

**Affiliations:** 1 Department of Mathematics, COMSATS University Islamabad, Attock Campus, Attock, 43600 Pakistan; 2 Future Technology Research Center, National Yunlin University of Science and Technology, 123 University Road, Section 3, Douliou, Yunlin 64002 Taiwan, ROC; 3 Department of Mathematics, College of Sciences, King Khalid University, Abha, 61413 Saudi Arabia; 4 Department of Mathematics, College of Arts and Sciences, Prince Sattam Bin Abdulaziz University, Wadi Aldawaser, 11991 Saudi Arabia; 5 Department of Clinical Laboratory Sciences, College of Applied Medical Sciences, Taif University, P.O. Box 11099, Taif, 21944 Saudi Arabia

**Keywords:** Mathematics and computing, Applied mathematics, Computational science

## Abstract

In current study, analysis is presented for peristaltic motion of applied magnetic field and entropy generation within couple stress (Cu/H_2_O) nanofluid through an endoscope. An endoscope contains two coaxial cylindrical tubes in which the internal tube is nonflexible while the external tube has sinusoidal wave passing through the boundary. Influences of mixed convection along with applied magnetic field are encountered as well. Formulated governing model is fabricated introducing long wavelength and creeping Stokesian flow approximation which are then analyzed numerically by utilizing Adams Bashforth method. For a physical insight, results are demonstrated to examine the behaviors of flow profiles and entropy generation number for emerging flow parameters with the help of graphs, bar-charts and tables.

## Introduction

Researchers have gained much attention in non-Newtonian fluid behaviours due to its novel applications in physiology, industry, and technological processes. Non-Newtonian fluids possess a nonlinear relationship among the rate of strain and shear stresses. Among the theories of non-Newtonian fluids, the couple stress fluid theory is an important one which is further a subclass of polar fluid theories introduced by Stokes. Constitutive relation that describes the behaviour of couple stress fluids encounter couple stresses along with classical Cauchy stress. Moreover, it is an oversimplification of the conventional theory of Newtonian fluids, which validates polar effects. Such fluids include biological fluids, cosmetics, slurries and dairy wastes etc. Characteristically, Devakar and Iyengar^1^ has been investigated flow dynamics of couple stress fluid configured inside two parallel plates. Geometries of the cylindrical pipes with slip wall conditions and analysis of couple stress fluid transport between the parallel surfaces have been obtained by Devakar et al.^2,3^. Srivastava^4^ analyzed consequences of axially symmetric mild stenosis for blood transport presuming blood as couple stress fluid. In order to inspect the performance of rheological complex fluids, investigations pertaining couple stress fluid are incredibly constructive^5–10^.

Furthermore, since several realistic fluids serve as couple stress fluids and their remarkable applications in heat transfer fields, thermal characteristics can be amplified by suspending particles with nanometer-size called nanoparticles pioneered by Choi^11^. For instance, Khan et al.^12^ have been investigated couple stress nanofluid flow through an oscillatory stretching sheet presuming the impacts of mixed convection with heat generation/absorption. Some remarkable applications regarding couple stress nanofluids are^13–16^.

Peristaltic motion has extensive applications in engineering processes, physiology and industry. In many biological systems, peristalsis has become one of the major apparatus for fluid transport, initially investigated by Engelman^17^. Recently, Hayat et al.^18^ have been investigated the impacts of convective conditions and nanoparticles on the peristaltic transport simultaneously. Moreover, the endoscope has many clinical applications. For medical recognition, the endoscope/annulus has important effects on the peristaltic flow. In cancer therapy, for desirable tissues removal, heat transfer is very extensively applicable. For instance, heat transfer in peristaltic flow through a vertical porous annulus has been presented by Vajravela et al.^19^. The closed-form solution of a nanofluid for the peristalsis in an annular section has been presented by Shahzadi and Nadeem^20^. Entropy generation analysis in the peristalsis of nanofluids due to complex flow structures has motivated the researchers. Entropy production in peristaltically occurred nanofluid flow has been analyzed by Hayat et al.^21^. The generation of entropy for couple stress fluid has been studied by Jangli et al.^22^. Further studies for fluid flows with entropy generation analysis can be seen in references^23–25^.

Magnetohydrodynamic explains the magnetic aspects of electrically conducting fluid and have numerous important usages in controlling the velocity of fluids by implementing magnetic field effects. Recently, Awan et al.^26^ inspected an unsteady hydro-magnetic nanofluid flow and heat transfer numerically through the channel. Simulation of computational fluid dynamics for suspension of nanoparticles in MHD liquid has been analyzed by Nawaz et al.^27^. Some ongoing research can be seen through the references^28–30^.

By utilizing the knowledge of pre-mentioned literature, the aim of current research is:

- To solve numerically the flow and heat exchange for couple stress nanofluid with entropy generation in existence of applied magnetic field and viscous dissipation.
- Formulated governing model is fabricated introducing long wavelength and creeping Stokesian flow approximation which are then analyzed numerically by utilizing Adams Bashforth method.
- Results are demonstrated to examine the behaviors of flow profiles and entropy generation number for emerging flow parameters with the help of graphs, bar-charts and tables.

## Problem development and governing model

**Figure 1:**
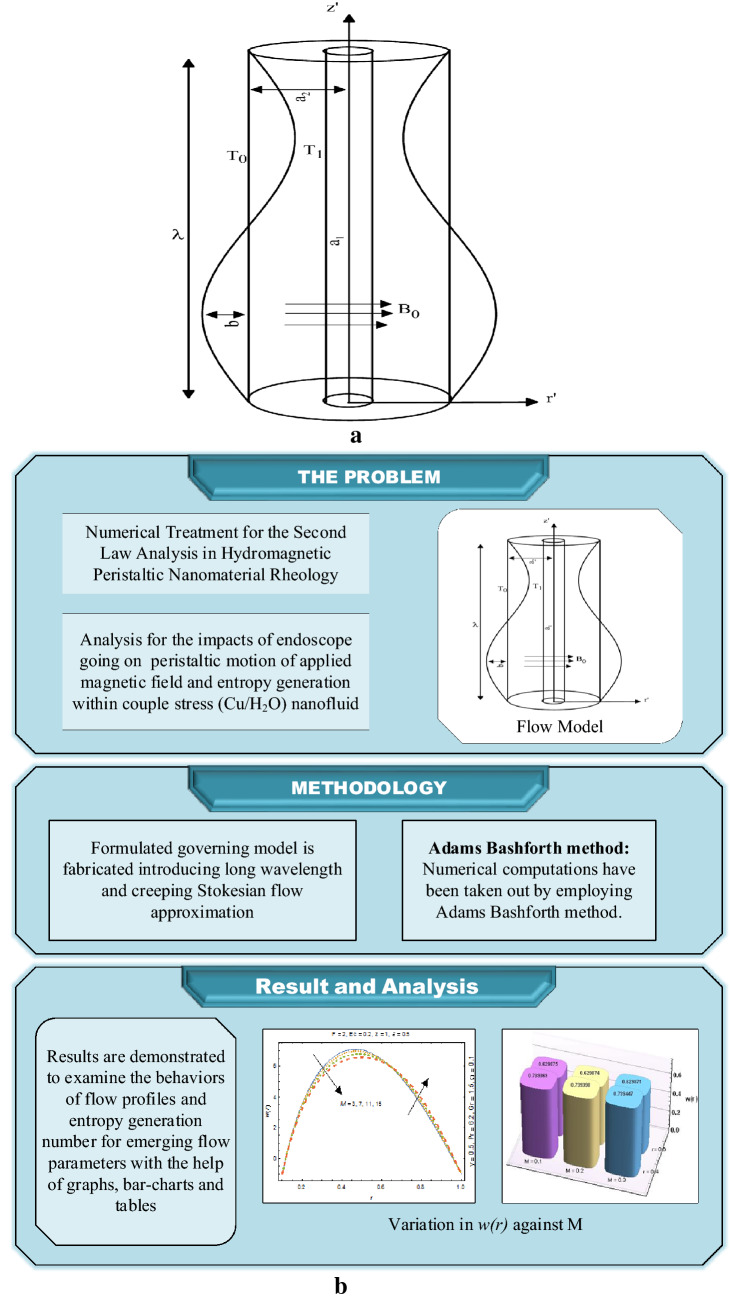
(**a**) Geometrical representation of flow problem. (**b**) Process flow architecture.

Assume the peristaltic motion of incompressible couple stress (Cu/water) nanofluids conducted in an endoscope. Along the tube wall, sinusoidal wave is transmitting with a uniform speed *c*. Cylindrical coordinate structure (*R, Z*) is preferred where *Z*-axis is passing through the central line and *R* indicates the radial direction as depicted in Fig. 1a, whereas the completer flow chart architecture is represented via Fig. 1b. For $a_{1}$ and $a_{2}$ being radii of internal and external tubes in endoscope, wave forms in fixed frame are written as^31,32^:

1 $$ r_{1} = a_{1} , $$

2 $$ r_{2} = a_{2} + b\sin \frac{2\pi }{\lambda }\left( {Z - ct} \right). $$

In which $\lambda$ is the wavelength, $b$ is the amplitude of wave and $t$ represents time of travelling wave. Moreover, *T*_1_ and *T*_0_ denote temperature of internal and external cylinders, accordingly.

The constitutive equations governing couple stress tensor $M$ and stress tensor $\tau$ are formulated as^7,8,32^:

3 $$ \tau = \left( { - P + \lambda_{1} {\nabla }{. }\overline{q} } \right)I +\upmu \left[ {{\nabla }\overline{q} + ({\nabla }\overline{q} )^{T} } \right] + \frac{1}{2}I{ \times }\left( {{\nabla }.M + \rho C} \right), $$

4 $$ M = mI + 2\eta {\nabla }\left( {{\nabla \times }\overline{q} } \right) + 2\eta^{{ / }} \left( {{\nabla }\left( {{\nabla \times }\overline{q} } \right)} \right)^{T} . $$

In which, $m$ denotes the $\frac{1}{3}$ trace of $M$, $\upmu$ and $\lambda_{1}$ express coefficients of viscosity, $C$ indicates vector of body couple while $\eta$ and $\eta^{{ / }}$ stand for coefficients of couple stress viscosity. The inequalities constraints for these material constants are given as:

5 $$\upmu \ge 0,{ 3}\lambda_{1} + 2\upmu \ge {0, }\eta \ge {0, }\left| {\eta^{{ / }} } \right| \le \eta . $$

In view of the above relations (3) and (4), the simplified equations in fixed frame of reference are^36^:

6 $$ \frac{\partial U}{{\partial R}} + \frac{U}{R} + \frac{\partial W}{{\partial Z}} = 0, $$

7 $$\uprho _{nf} \left( {\frac{\partial U}{{\partial t}} + U\frac{\partial U}{{\partial R}} + W\frac{\partial U}{{\partial Z}}} \right) = - \frac{\partial P}{{\partial R}} +\upmu _{nf} \left( {\frac{{\partial^{2} U}}{{\partial R^{2} }} + \frac{1}{R}\frac{\partial U}{{\partial R}} - \frac{U}{{R^{2} }} + \frac{{\partial^{2} U}}{{\partial Z^{2} }}} \right) - \eta \left( {\frac{{\partial^{4} U}}{{\partial R^{4} }} + 2\frac{{\partial^{4} U}}{{\partial R^{2} \partial Z^{2} }}} \right.\left. { + \frac{{\partial^{4} U}}{{\partial Z^{4} }} + \frac{2}{R}\frac{{\partial^{3} U}}{{\partial R^{3} }} + \frac{2}{R}\frac{{\partial^{3} U}}{{\partial R\partial Z^{2} }} - \frac{3}{{R^{2} }}\frac{{\partial^{2} U}}{{\partial R^{2} }} - \frac{2}{{R^{2} }}\frac{{\partial^{2} U}}{{\partial Z^{2} }} + \frac{3}{{R^{3} }}\frac{\partial U}{{\partial R}} - \frac{3}{{R^{4} }}U} \right), $$

8 $$ \rho_{nf} \left( {\frac{\partial W}{{\partial t}} + U\frac{\partial W}{{\partial R}} + W\frac{\partial W}{{\partial Z}}} \right) = - \frac{\partial P}{{\partial Z}} +\upmu _{nf} \left( {\frac{{\partial^{2} W}}{{\partial R^{2} }} + \frac{1}{R}\frac{\partial W}{{\partial R}} + \frac{{\partial^{2} W}}{{\partial Z^{2} }}} \right) - \eta \left( {\frac{{\partial^{4} W}}{{\partial R^{4} }} + \frac{{\partial^{4} W}}{{\partial R^{2} \partial Z^{2} }}} \right.\left. { + \frac{{\partial^{4} W}}{{\partial Z^{4} }} + \frac{2}{R}\frac{{\partial^{3} W}}{{\partial R^{3} }} + \frac{2}{R}\frac{{\partial^{3} W}}{{\partial R\partial Z^{2} }} - \frac{1}{{R^{2} }}\frac{{\partial^{2} U}}{{\partial R^{2} }} + \frac{1}{{R^{3} }}\frac{\partial W}{{\partial R}}} \right) + g\left( {\rho \beta_{T} } \right)_{nf} \left( {{{\rm T} - {\rm T}}_{0} } \right){ - \sigma }_{nf} B_{0}^{2} W, $$

9 $$ \left( {\rho c_{p} } \right)_{nf} \left( {\frac{\partial T}{{\partial t}} + U\frac{\partial T}{{\partial R}} + W\frac{\partial T}{{\partial Z}}} \right) = k^{*}_{nf} \left( {\frac{{\partial^{2} T}}{{\partial R^{2} }} + \frac{1}{R}\frac{\partial T}{{\partial R}} + \frac{{\partial^{2} T}}{{\partial Z^{2} }}} \right) + Q_{0} { + \Phi }{.} $$

In aforementioned model, *P* and *T* represent temperature and pressure of fluid whereas *U* and *W* express the *R* and *Z* components of velocity, respectively. Further, $g$ expresses gravitational acceleration, $B_{0}$ indicates intensity of external applied magnetic field, $\Phi$ denotes dissipation function and $Q_{0}$ is heat generation parameter.

In the laboratory frame (*R, Z*), flow is unsteady. In order to obtain a steady flow, transformations of quantities from the laboratory structure (*R, Z*) to the wave structure (r, z) are^7,32^:

10 $$ r = R,{\text{ z = Z}} - {\text{c}}t{\text{, u = U, w = W}} - {\text{c, p = P}}{. } $$

where, *u* and *v* denote the velocity components in the wave frame (*r, z*). Equations (5)–(8) yields:

11 $$ \frac{\partial u}{{\partial r}} + \frac{u}{r} + \frac{\partial w}{{\partial z}} = 0, $$

12 $$ \begin{gathered} A^{*} \left( {u\frac{\partial u}{{\partial r}} + \left( {w + c} \right)\frac{\partial u}{{\partial z}}} \right) = - \frac{1}{{\rho_{f} }}\frac{\partial p}{{\partial r}} + \frac{{\mu_{f} }}{{\rho_{f} }}\frac{1}{{A_{1} }}\left( {\frac{{\partial^{2} u}}{{\partial r^{2} }} + \frac{1}{r}\frac{\partial u}{{\partial r}} - \frac{u}{{r^{2} }} + \frac{{\partial^{2} u}}{{\partial z^{2} }}} \right) \hfill \\ - \eta \left( {\frac{{\partial^{4} u}}{{\partial r^{4} }} + 2\frac{{\partial^{4} u}}{{\partial r^{2} \partial z^{2} }} + \frac{{\partial^{4} u}}{{\partial z^{4} }} + \frac{2}{r}\frac{{\partial^{3} u}}{{\partial r^{3} }} + \frac{2}{r}\frac{{\partial^{3} u}}{{\partial r\partial z^{2} }} - \frac{3}{{r^{2} }}\frac{{\partial^{2} u}}{{\partial r^{2} }} - \frac{2}{{r^{2} }}\frac{{\partial^{2} u}}{{\partial z^{2} }} + \frac{3}{{r^{3} }}\frac{\partial u}{{\partial r}} - \frac{3}{{r^{4} }}u} \right), \hfill \\ \end{gathered} $$

13 $$ \begin{gathered} A^{*} \left( {u\frac{\partial w}{{\partial r}} + \left( {w + c} \right)\frac{\partial w}{{\partial z}}} \right) = - \frac{1}{{\rho_{f} }}\frac{\partial p}{{\partial z}} + \frac{{\mu_{f} }}{{\rho_{f} }}\frac{1}{{A_{1} }}\left( {\frac{{\partial^{2} w}}{{\partial r^{2} }} + \frac{1}{r}\frac{\partial w}{{\partial r}} + \frac{{\partial^{2} w}}{{\partial z^{2} }}} \right) \hfill \\ - \eta \left( {\frac{{\partial^{4} w}}{{\partial r^{4} }} + \frac{{\partial^{4} w}}{{\partial r^{2} \partial z^{2} }} + \frac{{\partial^{4} w}}{{\partial z^{4} }} + \frac{2}{r}\frac{{\partial^{3} w}}{{\partial r^{3} }} + \frac{2}{r}\frac{{\partial^{3} w}}{{\partial r\partial z^{2} }} - \frac{1}{{r^{2} }}\frac{{\partial^{2} u}}{{\partial r^{2} }} + \frac{1}{{r^{3} }}\frac{\partial w}{{\partial r}}} \right) \hfill \\ {\text{ + g}}\left( {\rho \beta_{T} } \right)_{f} A_{2} \left( {T - T_{0} } \right) + \sigma_{f} A_{4} B_{0}^{2} \left( {w + c} \right), \hfill \\ \end{gathered} $$

14 $$ A_{5} \left( {u\frac{\partial T}{{\partial r}} + \left( {w + c} \right)\frac{\partial T}{{\partial z}}} \right) = \frac{{k_{f} }}{{\left( {\rho c_{p} } \right)_{f} }}A_{3} \left( {\frac{{\partial^{2} T}}{{\partial r^{2} }} + \frac{1}{r}\frac{\partial T}{{\partial r}} + \frac{{\partial^{2} T}}{{\partial z^{2} }}} \right) + \frac{{Q_{0} }}{{\left( {\rho c_{p} } \right)_{f} }}{ + \Phi }{.} $$

where, constants involved in the above model are:

$$ \, A^{*} = \left( {1 -\upphi } \right) +\upphi \frac{{\rho_{s} }}{{\rho_{f} }},{\text{ A}}_{1} = \left( {1 -\upphi } \right)^{2.5} , \, A_{2} { = }\left( {{1 - }\upphi } \right) +\upphi \frac{{\left( {\rho \beta_{T} } \right)_{s} }}{{\left( {\rho \beta_{T} } \right)_{f} }}, $$

15 $$ A_{3} = \frac{{k_{s} + 2k_{f} - 2\upphi \left( {k_{f} - k_{s} } \right)}}{{k_{s} + 2k_{f} + 2\upphi \left( {k_{f} - k_{s} } \right)}}, \, A_{4} = \left( {1 -\upphi } \right) +\upphi \frac{{\sigma_{s} }}{{\sigma_{f} }}, \, A_{5} = \left( {1 -\upphi } \right) +\upphi \frac{{\left( {\rho c_{p} } \right)_{s} }}{{\left( {\rho c_{p} } \right)_{f} }}. $$

where $\upphi$ is volume fraction of nanoparticles whereas $\mu_{nf}$, $\rho_{nf}$, $(\beta_{T} )_{nf}$, $\upsigma _{nf}$ and $k^{*}_{nf}$ symbolizes the dynamic viscosity, density, thermal expansion coefficient, electric conductivity and thermal conductivity of nanofluid. Moreover, $\mu_{nf}$, $\rho_{s}$, $(\beta_{T} )_{s}$, $\upsigma _{s}$ and $k_{s}$ are dynamic viscosity, density, thermal expansion coefficient, electric conductivity and thermal conductivity of nanoparticles while $\mu_{f}$, $\rho_{f}$, $k_{f}$, $\upsigma _{f}$ and $(\beta_{T} )_{f}$ represents base liquid viscosity, density, thermal conductivity, electric conductivity and thermal expansion coefficient, respectively. Now, introducing dimensionless variables as^35^:

$$ \, \overline{r} { = }\frac{r}{{a_{2} }}, \, \overline{{r_{1} }} { = }\frac{{r_{1} }}{{a_{2} }} = c{ < }1, \, \overline{{r_{2} }} { = }\frac{{r_{2} }}{{a_{2} }} = 1 +\upphi \sin (2\pi z), \, \overline{u} = \frac{\lambda }{{a_{2} c}}u, \, \overline{z} { = }\frac{z}{\lambda }{, }\upphi = \frac{b}{{a_{2} }}{ < }1, $$

$$ \overline{w} = \frac{w}{c}, \, \delta = \frac{{a_{2} }}{\lambda }, \, \overline{t} { = }\frac{ct}{\lambda },{\text{ Re = }}\frac{{\rho_{f} ca_{2} }}{{\mu_{f} }}, \, \upsilon = \, \frac{{\mu_{f} }}{{\rho_{f} }},Gr{ = }\frac{{\rho_{f} g\left( {\beta_{T} } \right)_{f} a_{2}^{2} \left( {T_{1} - T_{0} } \right)}}{{\mu_{f} c}}, $$

$$ \overline{p} = \frac{{a_{2}^{2} }}{{\lambda \mu_{f} c}}p, \, \beta { = }\frac{{Q_{0} a_{2}^{2} }}{{k_{f} \left( {T_{1} - T_{0} } \right)}}, \, \theta { = }\frac{{\left( {T - T_{0} } \right)}}{{\left( {T_{1} - T_{0} } \right)}},{\text{ y = }}\sqrt {\frac{\eta }{{\mu_{f} a_{2}^{2} }}} ,{\text{ Pr = }}\frac{{\mu_{f} \left( {c_{p} } \right)_{f} }}{{k_{f} }}, $$

16 $$ \, \overline{\psi } = \frac{\psi }{{a_{2} c}},\overline{u} = - \frac{\delta }{{\overline{r}}}\frac{{\partial \underset{\raise0.3em\hbox{$\smash{\scriptscriptstyle\cdot}$}}{\psi } }}{{\partial \overline{z}}},\overline{w} = \frac{1}{{\overline{r}}}\frac{{\partial \overline{\psi }}}{{\partial \overline{r}}},{\text{Ec = }}\frac{{c^{2} }}{{\left( {c_{p} } \right)_{f} \left( {T_{1} - T_{0} } \right)}},{\text{ M}}^{2} { = }\frac{{\sigma_{f} B_{0}^{2} a_{2}^{2} }}{{\mu_{f} }}. $$

With the help of long wavelength and creeping flow approximations Eq. (11) is identically fulfilled while Eqs. (12)–(14) reduces to the following expressions:

17 $$ \frac{\partial p}{{\partial r}} = 0, $$

18 $$ y^{2} \left( {\frac{{\partial^{2} }}{{\partial r^{2} }} + \frac{1}{r}\frac{\partial }{\partial r}} \right)^{2} \left( {\frac{1}{r}\frac{\partial \psi }{{\partial r}}} \right) - \frac{1}{{A_{1} }}\left( {\frac{{\partial^{2} }}{{\partial r^{2} }} + \frac{1}{r}\frac{\partial }{\partial r}} \right)\left( {\frac{1}{r}\frac{\partial \psi }{{\partial r}}} \right) = - \frac{1}{{A_{1} }}\frac{\partial p}{{\partial z}} + A_{2} Gr\theta - A_{4} M^{2} \left( {\frac{1}{r}\frac{\partial \psi }{{\partial r}} + 1} \right), $$

By differentiating Eq. (18) with respect to *r*, we get the following expression:

19 $$ \begin{gathered} y^{2} \frac{\partial }{\partial r}\left( {\frac{{\partial^{2} }}{{\partial r^{2} }} + \frac{1}{r}\frac{\partial }{\partial r}} \right)^{2} \left( {\frac{1}{r}\frac{\partial \psi }{{\partial r}}} \right) - \frac{1}{{A_{1} }}\frac{\partial }{\partial r}\left( {\frac{{\partial^{2} }}{{\partial r^{2} }} + \frac{1}{r}\frac{\partial }{\partial r}} \right)\left( {\frac{1}{r}\frac{\partial \psi }{{\partial r}}} \right) - A_{2} Gr\frac{\partial \theta }{{\partial r}} + \hfill \\ A_{4} M^{2} \frac{\partial }{\partial r}\left( {\frac{1}{r}\frac{\partial \psi }{{\partial r}} + 1} \right) = 0, \hfill \\ \end{gathered} $$

20 $$ A_{3} \left( {\frac{{\partial^{2} \theta }}{{\partial r^{2} }} + \frac{1}{r}\frac{\partial \theta }{{\partial r}}} \right) + \beta + A_{1} Ec\Pr \left( {\frac{ - 1}{{r^{2} }}\frac{\partial \psi }{{\partial r}} + \frac{1}{r}\frac{{\partial^{2} \psi }}{{\partial r^{2} }}} \right)^{2} = 0. $$

Bar notation is ignored. Here, *Re, Ec*, *Pr* and *Gr* indicates Reynolds number, Eckert number, Prandtl number and thermal Grashof number whereas $\delta$ expresses wave number and $\theta$ is the dimensionless temperature. Moreover, $\psi$ is stream function for which components of velocity are derived as:

$$ u = \frac{1}{r}\frac{\partial \psi }{{\partial r}},v = - \frac{\delta }{r}\frac{\partial \psi }{{\partial z}}. $$

The dimensionless boundary conditions are:

21 $$ \frac{1}{r}\frac{\partial \psi }{{\partial r}}{ = - 1, }\frac{{\partial^{2} }}{{\partial r^{2} }}\left( {\frac{1}{r}\frac{\partial \psi }{{\partial r}}} \right) = 0, \, \psi = \, \frac{ - F}{2}{, }\theta {\text{ = 1, at r = r}}_{1} { ,} $$

22 $$ \frac{1}{r}\frac{\partial \psi }{{\partial r}}{ = - 1, }\frac{{\partial^{2} }}{{\partial r^{2} }}\left( {\frac{1}{r}\frac{\partial \psi }{{\partial r}}} \right) = 0, \, \psi = \, \frac{F}{2}{, }\theta {\text{ = 0, at r = r}}_{2} \, {.} $$

## Analysis of entropy generation

Entropy is the measure of molecular disorder. The volumetric entropy generation rate can be expressed as^31^:

23 $$ \dot{S}^{{\prime\prime\prime}}_{Gen} = \frac{{\kappa_{nf} }}{{T_{0}^{2} }}\left( {\frac{\partial T}{{\partial R}}} \right)^{2} + \frac{{\mu_{nf} }}{{T_{0} }}\left( {\frac{\partial W}{{\partial R}}} \right)^{2} + \frac{{\sigma B_{0}^{2} W^{2} }}{{T_{0} }}. $$

Moreover, characteristic entropy generation rate obtained by using boundary conditions in Eq. (23) is:

24 $$ \dot{S}^{{\prime\prime\prime}}_{0} = \frac{{\kappa_{f} }}{{T_{0}^{2} a_{2}^{2} }}\left( {T_{1} - T_{0} } \right)^{2} , $$

By using Eq. (24) and dimensionless variables in Eq. (23), dimensionless entropy generation rate is:

25 $$ N_{s} = \frac{{\dot{S}^{{\prime\prime\prime}}_{Gen} }}{{\dot{S}^{{\prime\prime\prime}}_{0} }} = A_{3} \left( {\frac{\partial \theta }{{\partial r}}} \right)^{2} + \varepsilon Ec\Pr \frac{1}{{A_{1} }}\left( {\frac{ - 1}{{r^{2} }}\frac{\partial \psi }{{\partial r}} + \frac{1}{r}\frac{{\partial^{2} \psi }}{{\partial r^{2} }}} \right)^{2} + \varepsilon Ec\Pr M^{2} \left( {\frac{1}{r}\frac{\partial \psi }{{\partial r}}} \right)^{2} . $$

where, $\varepsilon = \frac{{T_{0} }}{{\left( {T_{1} - T_{0} } \right)}}$ represents temperature difference parameter.

## Discussion of results

In this section, effects of important parameters on the velocity and temperature with dimensionless entropy generation number are portrayed graphically. Numerical computations have been taken out by employing Adams Bash forth method that is an iterative technique. The Adams Bash forth methods allow us to compute the approximate solution at a given instant from prior instants' solutions explicitly.

**Figure 2:**
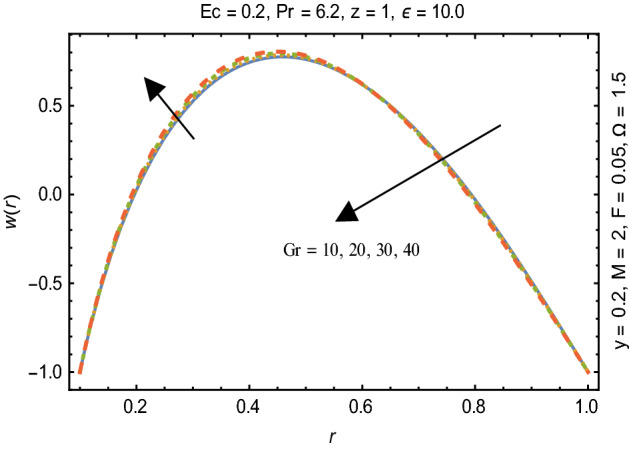
Deviation in *w(r)* against G*r*.

**Figure 3:**
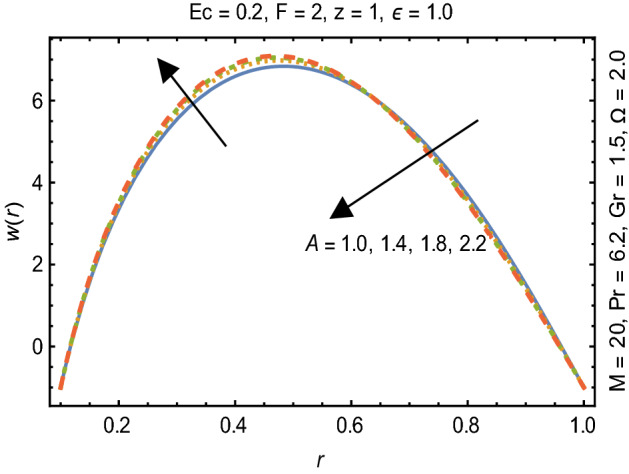
Deviation of *w(r*) against y$.$

**Figure 4:**
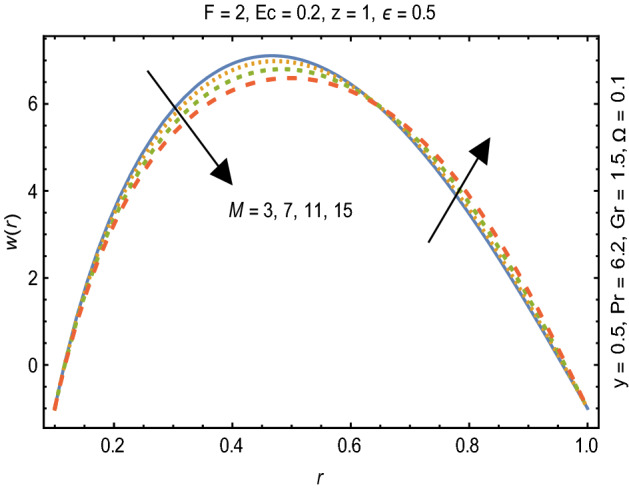
Variation in *w(r)* against M.

**Figure 5:**
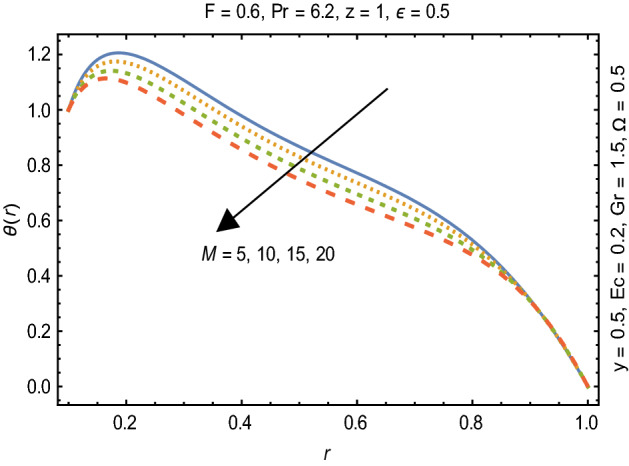
Variation in $\theta$*(r)* against M.

**Figure 6:**
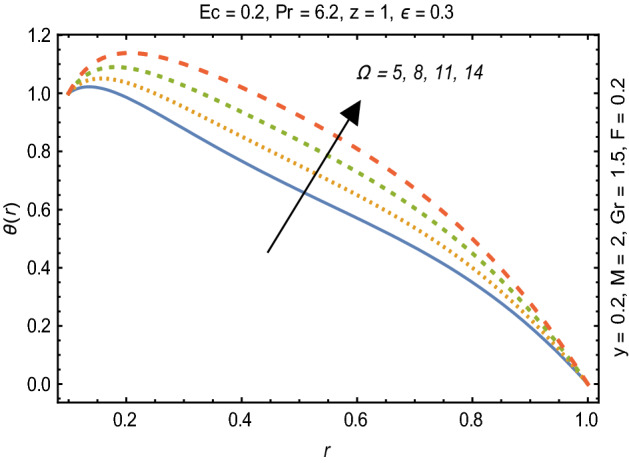
Variation in $\theta$*(r)* against $\Omega$_._

**Figure 7:**
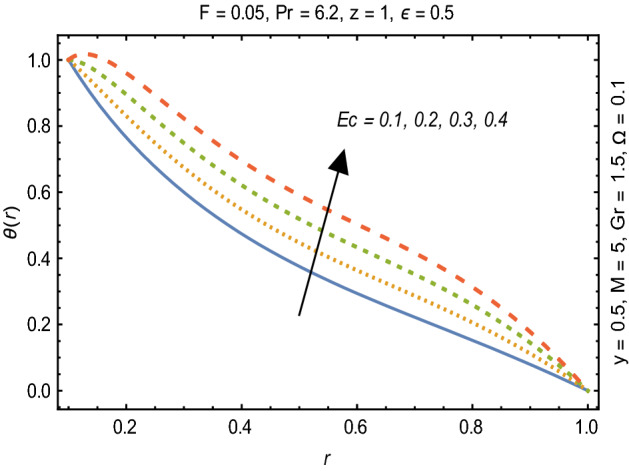
Variation in $\theta$*(r)* against Ec.

**Figure 8:**
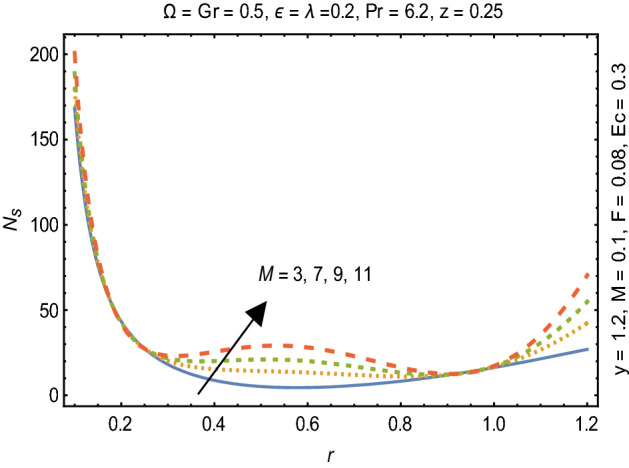
Variation in $N_{s}$ for M.

**Figure 9:**
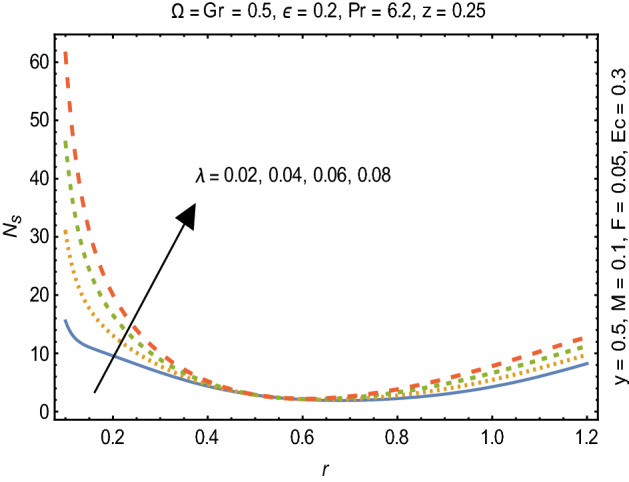
Variation in $N_{s}$ for $\lambda$.

**Figure 10:**
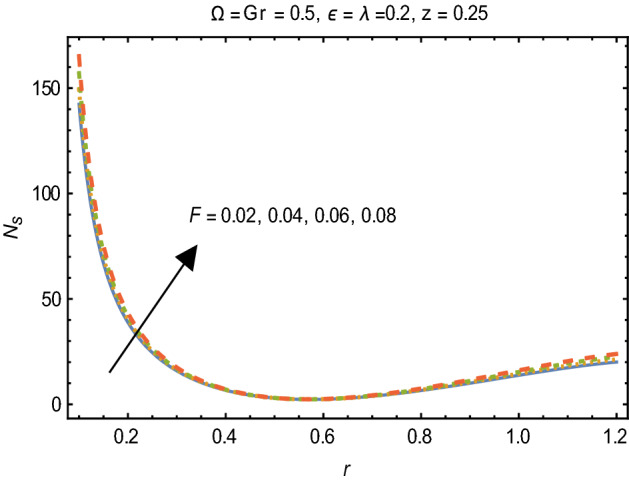
Variation in $N_{s}$ for F.

**Figure 11:**
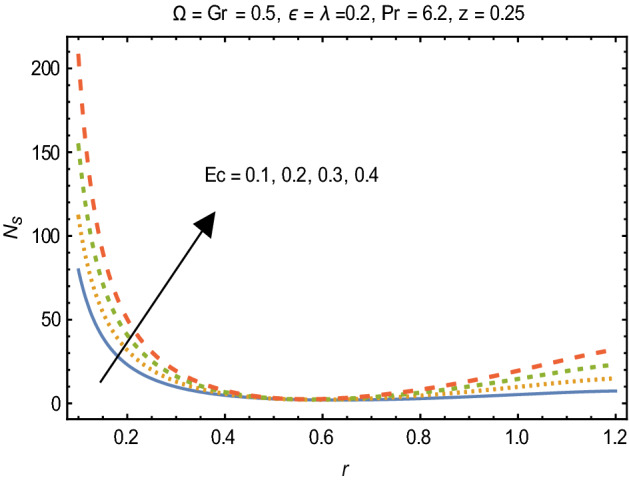
Variation in $N_{s}$ for *Ec*.

Figures 2 and 3 portray the behavior of velocity toward rising magnitude of velocity parameter *y* and Grashof number *G*r. It is noticed that for rising values of both parameters, *w(r*) augments close the endoscope and declines in the vicinity of the tube walls. The buoyancy forces play a leading effect close to the endoscope and hence fluid velocity upgrades as one move close to the endoscopic tube. Moreover, viscous forces are more dominant near the peristaltic tube so flow rate tends to decrease. Figure 4 explored the oscillatory behavior of *w(r*) for several values of magnetic parameters *M.* Enlargement in velocity of the fluid near the endoscope is due to dominant effects of Lorentz force which slowly reduces near the tube wall because of no slip condition. Variational trend of temperature of the fluid is explored in Figs. 5, 6, and 7. Increment in magnitude of magnetic parameter causes temperature of the entire fluid to decrease as plotted in Fig. 5. Physics behind such behavior is increasing resistive effects of Lorentz force. Figures 6 and 7 reveal that temperature increases for rising values of $\Omega$ and *Ec.* Higher values of $\Omega$ yield heat generation while increment in *Ec* leads to higher kinetic energy due to which temperature of the fluid rises. Behavior of entropy generation number *Ns* towards physical parameters is illustrated graphically and plotted in Figs. 8, 9, 10, and 11. Magnitude of entropy generation number towards *M* is increasing near the walls and in the central region including points of intersection at which entropy remains constant. This trend is due to enhancing frictional effects of Lorentz force (Fig. 8).

Figure 9 explicates that higher magnitude of temperature difference parameter has an impact of increasing irreversibility. Physically, it is due to high temperature gradient close to the boundaries. A similar behavior of *Ns* for *F* and *Ec* is observed from Figs. 10 and 11 that are caused by no slip wall conditions and thus large velocity gradients.

Further, numerical values for thermophysical properties of nanofluid along with empirical formulas are presented in Tables 1 and 2. Tabulated observations of velocity and temperature against rising values of nondimensional parameters are displayed in Tables 3 and 4. Moreover, bar charts are drawn for a detailed view. Both of the tabulated as well as bar chart view reveal that with the variations in *Gr* and *M*, magnitude of *w(r)* increases at *r* = 0.4 (near endoscope) and decreases at *r* = 0.6 (near tube). These behaviors clearly satisfied the graphical results. Except this*, θ(r)* rises for higher magnitudes of Eckert number and magnetic parameter.

**Table 1:** Empirical values of thermophysical features for base fluid (water) and nanoparticles (copper)^23^.

Properties/constituents	H_2_O	Cu
Density, ρ(Kg/m^3^)	997	8933
Specific heat, C_p_ (J/kg K)	4179	385
Thermal conductivity, κ (W/m K)	0.613	401
Thermal expansion coefficient, β(10^−6^ m/(mK))	210	16.65
Electrical conductivity, σ (S/m)	0.05	5.96 × 10^7^

**Table 2:** Expressions for thermophysical features for nanoparticles (copper).

Properties	Nanofluid
Density	$\rho_{nf} = \rho_{f} \left[ {\left( {1 -\upphi } \right) +\upphi \frac{{\rho_{s} }}{{\rho_{f} }}} \right]$
Heat capacity	$\left( {\rho c_{p} } \right)_{nf} = \left( {\rho c_{p} } \right)_{f} \left[ {\left( {1 -\upphi } \right) +\upphi \frac{{\left( {\rho c_{p} } \right)_{s} }}{{\left( {\rho c_{p} } \right)_{f} }}} \right]$
Viscosity	$\mu_{nf} = \frac{{\mu_{f} }}{{\left( {1 -\upphi } \right)^{2.5} }}$
Thermal conductivity	$\frac{{k^{*}_{nf} }}{{k_{f} }} = \frac{{k_{s} + 2k_{f} - 2\upphi \left( {k_{f} - k_{s} } \right)}}{{k_{s} + 2k_{f} + 2\upphi \left( {k_{f} - k_{s} } \right)}}$
Thermal expansion coefficient	$\left( {\rho \beta_{T} } \right)_{nf} { = }\left( {\rho \beta_{T} } \right)_{f} \left[ {\left( {{1} -\upphi } \right) +\upphi \frac{{\left( {\rho \beta_{T} } \right)_{s} }}{{\left( {\rho \beta_{T} } \right)_{f} }}} \right]$
Electric conductivity	$\sigma_{nf} = \sigma_{f} \left[ {\left( {1 -\upphi } \right) +\upphi \frac{{\sigma_{s} }}{{\sigma_{f} }}} \right]$

**Table 3:** Results for velocity profile against nondimensional parameters.

Cu/H_2_o							
M	F	Ec	Gr	w (r = 0.2)	w (r = 0.4)	w (r = 0.6)	w (r = 0.8)
0.1	0.2	0.2	0.1	0.003951	0.739369	0.629875	–0.028079
0.2				0.003980	0.739398	0.629874	–0.028096
0.3				0.004029	0.739447	0.629871	–0.028125
	0.2			0.280305	1.21785	1.07855	0.239451
	0.4			0.648593	1.85599	1.67672	0.596190
	0.6			1.01689	2.49417	2.2749	0.952926
		0.2		0.003951	2.49425	2.27477	–0.028079
		0.4		0.003930	2.49451	2.2748	–0.028063
		0.6		0.003909	2.49477	2.27482	–0.028047
			0.1	0.002273	0.738773	0.629913	–0.021832
			0.2	0.003137	0.738816	0.62991	–0.025778
			0.3	0.004566	0.738858	0.629908	–0.029528

**Table 4:** Results for temperature profile against nondimensional parameters.

M	F	Ec	Gr	θ (r = 0.2)	θ (r = 0.4)	θ (r = 0.6)
0.1	0.2	0.2	0.1	1.06589	0.544824	0.361133
0.2				1.06590	0.544827	0.361136
0.3				1.06591	0.544833	0.361142
	0.2			0.906249	1.27564	0.446693
	0.4			1.04157	1.8217	0.592958
	0.6			1.21094	2.50981	0.776021
		0.2		1.06589	0.544833	0.361142
		0.4		1.05017	0.688935	0.497805
		0.6		1.03445	0.833058	0.634488
			0.1	1.06081	0.544757	0.361070
			0.2	1.06407	0.544762	0.361075
			0.3	1.06699	0.544767	0.361079

**Figure 12:**
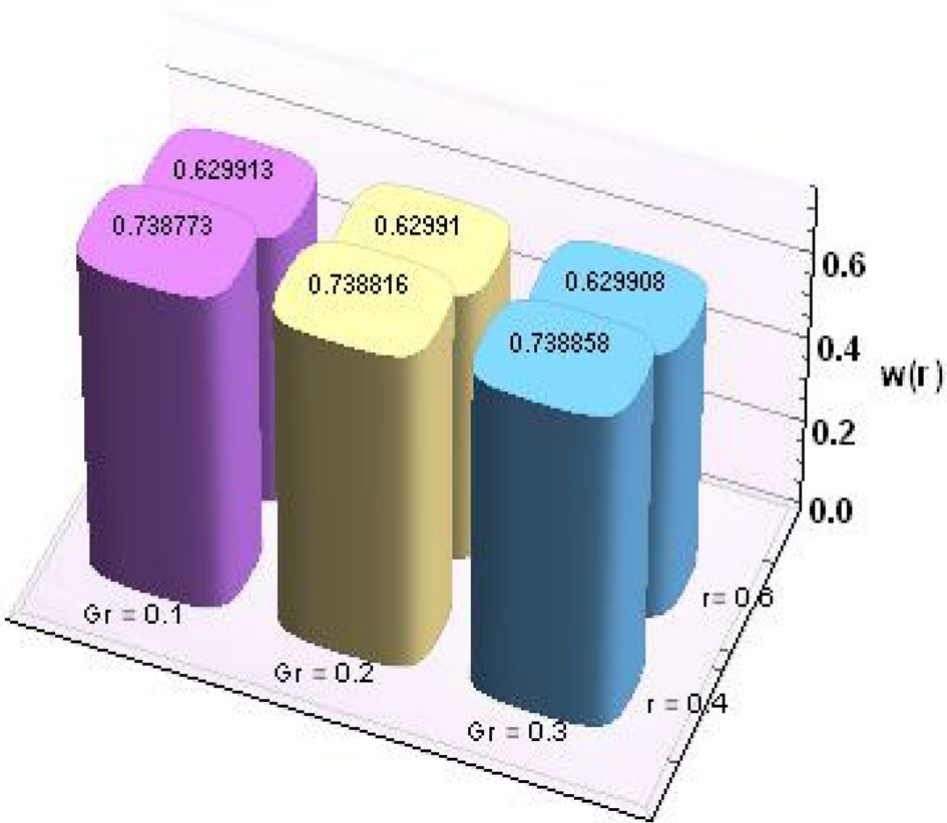
Variation in *w(r)* against *Gr*.

**Figure 13:**
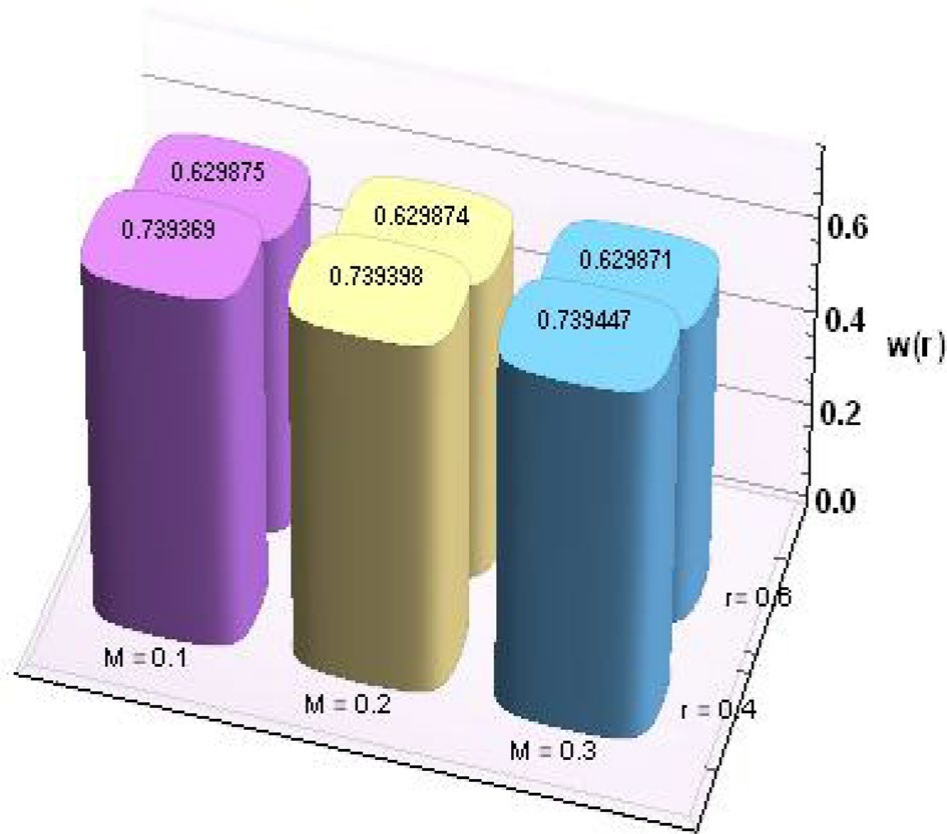
Variation in *w(r)* against *M*.

**Figure 14:**
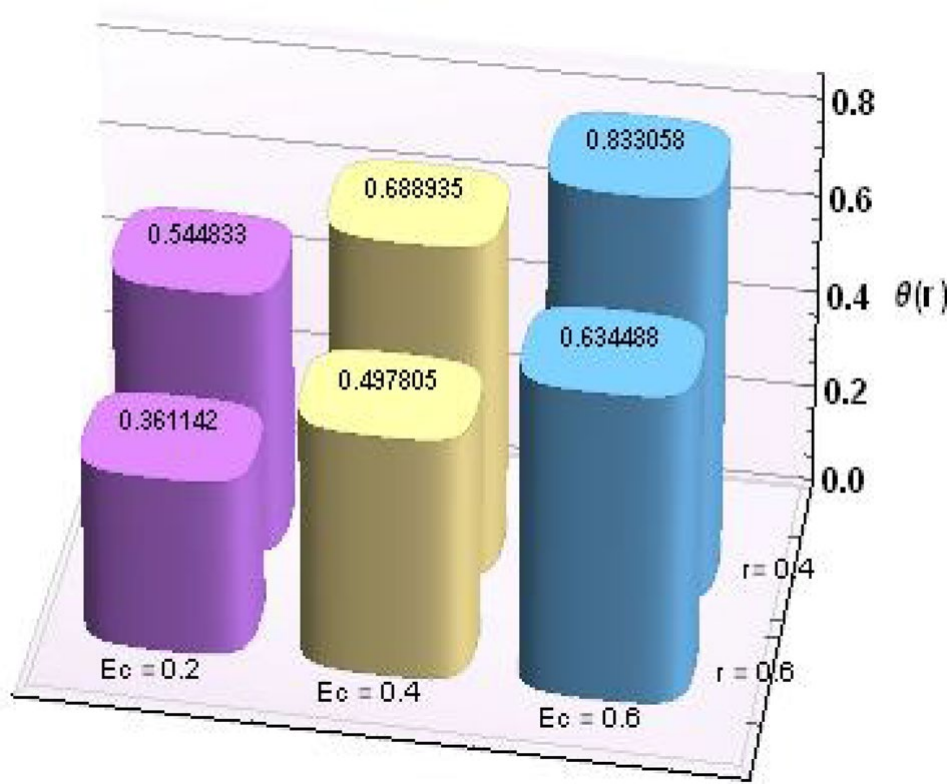
Variation in *θ(r)* against *Ec*.

**Figure 15:**
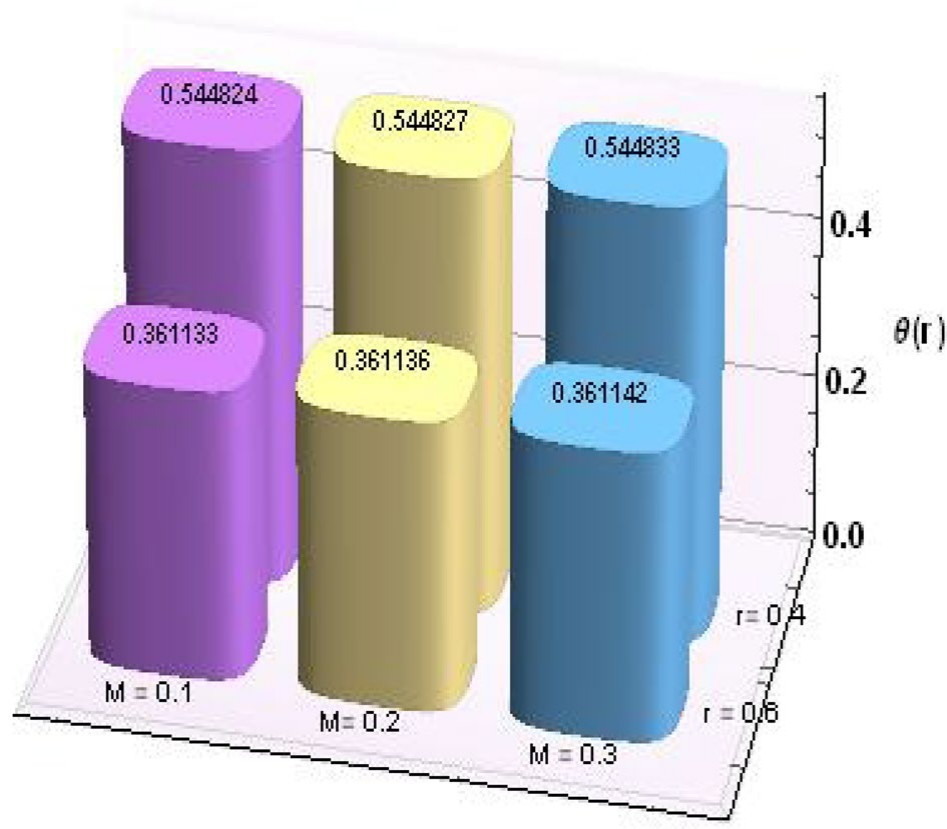
Variation in *θ(r)* against *M*.

**Figure 16:**
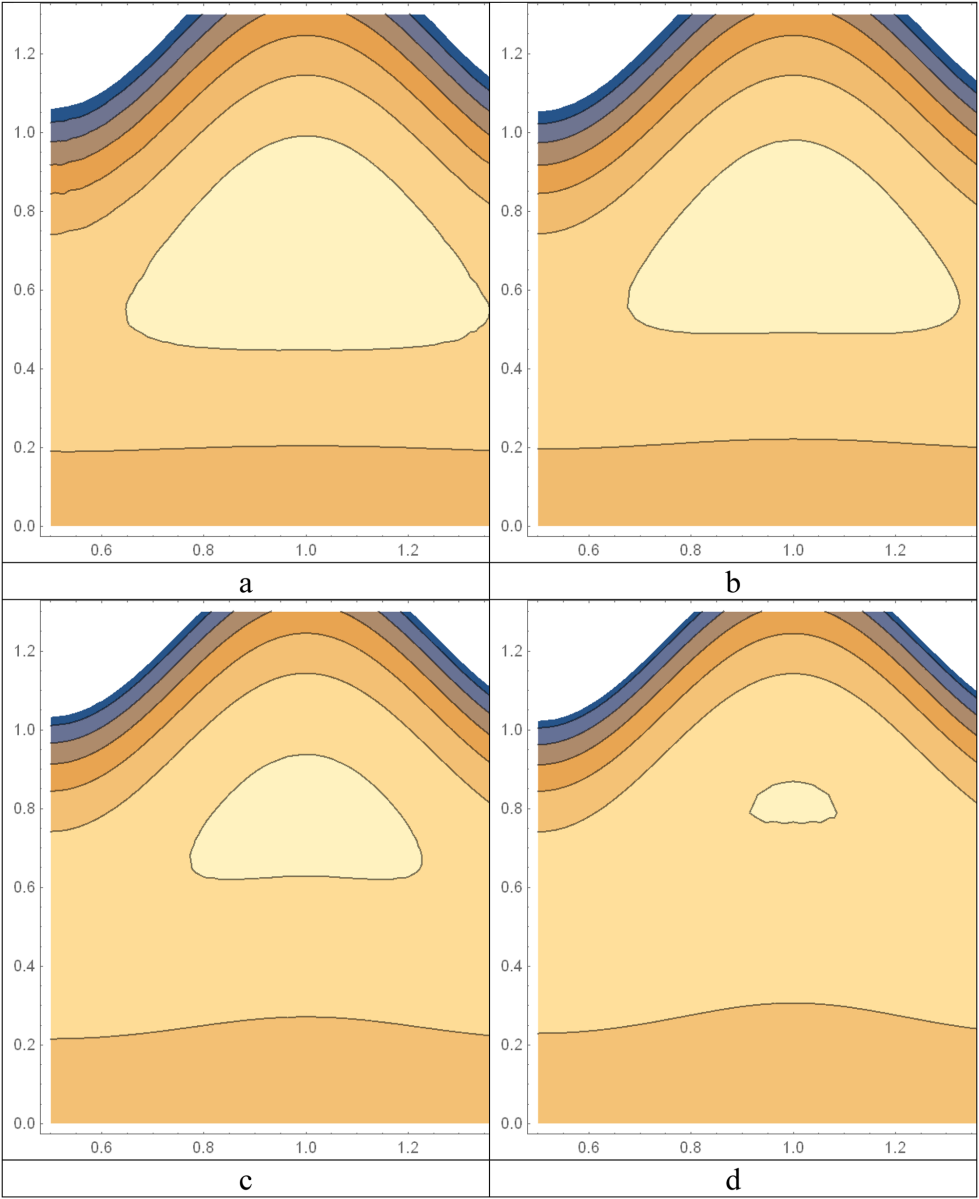
Streamlines for different M, (**a**) M = 0.01, (**b**) M = 1.0, (**c**) M = 2.0, (**d**) M = 3.0.

Figures 12 and 13 are prepared to present the bar-charts showing the influence of *w(r)* against *Gr* and *M* respectively. It is observed that for radial value *r* = 0.4 results are significant as compared to *r* = 0.6. Moreover Figs. 14 and 15 elucidate the effects of temperature profile *θ(r)* vs *Ec* and *M*. We noticed that temperature profile *θ(r)* signifies rapidly for larger values of Ec near the walls whereas temperature profile *θ(r)* has qualitatively similar behavior for different values of *M.* Figure 16 is constructed for the streamlines of the presented fluid flow corresponding to various parameters of interests associated the fluid flow system. The flow pattern is shown by the streamlines presented via Fig. 16.

## Concluding remarks

In the present research article, analysis for the impacts of endoscope going on peristaltic flow of couple stress nanofluid in the existence of magnetic field and viscous dissipation is carried out. Major conclusions drawn from present investigation are:

- Velocity profile increases close to the endoscope and decreases close to the peristaltic vertical tube with increment of *Gr* and *y* while an opposite behavior is noticed for *M*.
- Temperature increases against higher values of $\Omega$ and *Ec* but an opposite behavior is depicted for *M*.
- Entropy is directly affected by buoyancy and viscous forces which are dominant near the endoscope and tube walls.
- The consequences of Newtonian fluid model can be obtained by taking the couple stress parameter *y* = *0* within the current model.

## Future work

In the future research, the soft computing intelligent techniques can be implemented as an efficient/accurate stochastic numerical solver implemented in nonlinear computational fluid mechanics models^33–36^, singular and multi-singular differential systems^37–39^ and mathematical models representing the problems of epidemiology^40–42^.
